# Noninferiority Clinical Trial of Adapted START NOW Psychotherapy for Outpatient Opioid Treatment

**DOI:** 10.21203/rs.3.rs-3229052/v1

**Published:** 2023-08-11

**Authors:** Albert Truong, Anita Kablinger, Cheri Hartman, David Hartman, Jennifer West, Alexandra Hanlon, Alicia Lozano, Robert McNamara, Richard Seidel, Robert Trestman

**Affiliations:** Virginia Tech; Carilion Clinic; Carilion Clinic; Carilion Clinic; Virginia Tech; Virginia Tech; Virginia Tech; Carilion Clinic; Carilion Clinic; Carilion Clinic

**Keywords:** Opioid use disorder, office-based opioid treatment, psychotherapy, group therapy, medications for opioid use disorder

## Abstract

**Background:**

Medications for opioid use disorder (MOUD) such as buprenorphine is effective for treating opioid use disorder (OUD). START NOW (SN) is a manualized, skills-based group psychotherapy originally developed and validated for the correctional population and has been shown to result in reduced risk of disciplinary infractions and future psychiatric inpatient days with a dose response effect. We investigate whether adapted START NOW is effective for treating OUD in a MOUD office-based opioid treatment (OBOT) setting in this non-inferiority clinical trial.

**Methods:**

Patients enrolled in once weekly buprenorphine/suboxone MOUD OBOT were eligible for enrollment in this study. Participants were cluster-randomized, individually-randomized, or not randomized into either START NOW psychotherapy or treatment-as-usual (TAU) for 32 weeks of therapy. Treatment effectiveness was measured as the number of groups attended, treatment duration, intensity of attendance, and overall drug use as determined by drug screens.

**Results:**

137 participants were quasi-randomized to participate in SN (n = 79) or TAU (n = 58). Participants receiving START NOW psychotherapy, when compared to TAU, had comparable number of groups attended (16.5 vs. 16.7, p = 0.80), treatment duration in weeks (24.1 vs. 23.8, p = 0.62), and intensity defined by number of groups attended divided by the number of weeks to last group (0.71 vs. 0.71, p = 0.90). SN compared to TAU also had similar rates of any positive drug screen result (81.0% vs. 91.4%, p = 0.16). This suggests that adapted START NOW is noninferior to TAU, or the standard of care at our institution, for treating opioid use disorder.

**Conclusion:**

Adapted START NOW is an effective psychotherapy for treating OUD when paired with buprenorphine/naloxone in the outpatient group therapy setting. Always free and publicly available, START NOW psychotherapy, along with its clinician manual and training materials, are easily accessible and distributable and may be especially useful for low-resource settings in need of evidence-based psychotherapy.

## BACKGROUND

In 2019, 10.1 million people aged 12 or older in the United States misused opioids, accounting for 3.7% of the population (1). In the U.S., medications for opioid use disorder (MOUD) is subject to federal legislation and regulation including the Drug Addiction Treatment Act of 2000 and federal regulation 42 CFR 8. Under federal law, MOUD patients must be able to receive counseling, which may entail different forms of behavioral therapy with additional medical and social services. However, access to treatment is extremely limited in the U.S.; although 1.6 million Americans qualify for the diagnosis of OUD based on the *Diagnostic and Statistical Manual of Mental Disorders, Fifth Edition*, according to the Substance Abuse and Mental Health Services Administration (SAMHSA), only 18.1% of these individuals received MOUD in the past year (2).

Furthermore, SAMHSA, the American Society of Addiction Medicine (ASAM), and the World Health Organization (WHO) support MOUD due to its proven effectiveness for treating OUD (3–5). Yet none of these organizations have declared a specific form of behavioral therapy to be the most effective for treating OUD when paired with MOUD. In addition to improving access and expanding capacity to provide MOUD, randomized clinical trials are needed to determine which form of psychotherapy is most effective for OUD (6).

The treatment of OUD is complicated by psychiatric comorbidities, psychosocial challenges such as incarceration, and socioeconomic concerns such as unemployment and homelessness that affect individuals with opioid use disorder (7, 8). One study found that 47.1% of individuals with prescription opioid dependence were also diagnosed with comorbid mood or anxiety disorders (9). Furthermore, opioid misuse is related to other substance use and other psychiatric illnesses such as major depressive episodes (10). For example, in the U.S., 13.8% with serious mental illness and 8.8% with any mental illness misused opioids in the past year compared to just 2.5% of adults with no mental illness (1).

We hypothesize that integrated, comprehensive interventions are needed to effectively treat individuals with OUD due to these numerous, impairing comorbidities. Specifically, we refer to this holistic approach as an intervention that addresses the whole patient with regards to their medical, psychosocial, and socioeconomic well-being (11, 12). In our office-based opioid treatment (OBOT) setting, we propose that MOUD with buprenorphine/naloxone should be paired with a more holistic psychotherapy. We propose that such a comprehensive intervention is START NOW, a psychotherapy that was originally implemented and validated in the Connecticut Correctional Health Research Program with support from a National Institute of Justice grant (NIJ 2002-IJ-CX-K009) (13). For the purposes of treating substance use disorders (SUD), START NOW has since been modified and validated in a pilot study so that it is more appropriate and applicable for the OUD patient population (14).

START NOW is a manual-guided skills training psychotherapy that integrates cognitive behavior therapy, motivational interviewing, trauma-informed care, and elements of cognitive neuro-rehabilitation (13). Entirely free and available in the public domain, START NOW was originally designed for low-resource settings and as a psychotherapy for incarcerated individuals who present with mood dysregulation, impulsivity, aggression, and interpersonal discord. A retrospective cohort analysis of 850 patients in Connecticut state prison demonstrated a significantly reduced risk of disciplinary infractions and future psychiatric inpatient days with a dose response effect (15, 16). Furthermore, START NOW was associated with reduced risk of criminal recidivism in an evaluation of a specialized alternative-to-incarceration program for individuals with serious mental illness and co-occurring SUD (17). START NOW is currently in use in approximately 20 U.S. states and five countries in correctional facilities, forensic psychiatric hospitals, and community settings. Internationally, investigators at the University of Basel are evaluating START NOW in female adolescents with oppositional defiant disorder or conduct disorder (18). Their cluster-randomized, multi-center, controlled trial of 177 subjects over 12 weeks demonstrated a significant reduction in conduct/oppositional symptoms three months after the end of intervention with high satisfaction (19).

The purpose of this study was to assess non-inferiority of START NOW compared to treatment-as-usual (TAU) for treating the OUD patient population in an outpatient setting since equivalence or superiority has not yet been shown for either treatment. We hypothesize that adapted START NOW will be an effective treatment for OUD, which we explored in this hybrid cluster- and individually-randomized non-inferiority clinical trial. From here forward, we refer to this hybrid randomized design as simply quasi-randomized. We used this model to test the feasibility of empirically evaluating new psychotherapies in the real world with its inherent challenges when performing a true individually-randomized controlled trial was not feasibly possible. Treatment effectiveness was measured as the number of groups attended, treatment duration, intensity of attendance, and overall drug use as determined by drug screens. Showing that START NOW is effective for treating OUD will provide evidence for its utility in the clinical setting. START NOW has the potential to greatly benefit resource-limited settings because START NOW psychotherapy, along with its clinician manual and training materials, are always free, publicly available, and easily accessible.

## METHODS

### Participants:

Participants were eligible for enrollment if they were at least 18 years of age and enrolled in the outpatient medications for opioid use disorder (MOUD) with buprenorphine/naloxone (Suboxone, Indivior Inc.) office-based opioid treatment (OBOT) program. Exclusion criteria included participants unable to commit to the planned 32 session regimen, had an unmanaged psychiatric illness that would interfere with study participation (the ability to attend group therapy sessions), posed an imminent suicide risk–as assessed by the investigators–or were pregnant.

Research coordinators approached individuals who were under the care of clinicians in the OBOT program. The potential participants’ psychiatric clinicians had to agree with their patient’s involvement in the study. At our institution, all patients enrolled in the OBOT program are required to be involved in group therapy in addition to receiving MOUD. As a result, if patients were not amenable to group therapy, they were also not a candidate to participate in the OBOT program or this clinical trial.

### Study Design and Treatment:

In this quasi-randomized clinical trial, participants received either adapted START NOW psychotherapy for OUD or treatment-as-usual (TAU). The START NOW psychotherapy utilized for treating substance use disorder is publicly available at https://www.carilionclinic.org/start_now. Participants were either individually randomized into START NOW or TAU therapy, cluster randomized (randomized together with a group of other participants into either START NOW or TAU), or not randomized and simply assigned into a treatment condition. As a result, this study utilized a quasi-randomized clinical trial schema. Some group sessions were on days/times agreeable to the group participants’ schedules; as a result, groups–and therefore all associated participants in these groups–were cluster-randomized to either START NOW or TAU. This allowed for preservation of the original group’s day of the week and time. Brand new patients to the OBOT program were individually randomized to either SN or TAU groups. However, if the ability to attend therapy was limited to certain days or times due to travel or job confines, new participants were assigned to a specific group/day/time, limiting the ability to perform randomization.

All medical care occurred at a single medical center located in southwestern Virginia. Our institution does not have a standard of care psychotherapy for treating OUD. As a result, each group-based psychotherapy in the OBOT program was organized and led by psychiatric physicians and nurse practitioners, each utilizing a group-based psychotherapy based on their own professional discretion. This was the standard of practice at our institution prior to this study and persisted during our investigation. For the purposes of this study, we refer to these different psychotherapy groups as treatment-as-usual. These TAU groups serve as a control group compared to START NOW. Participants enrolled in the START NOW cohort were only treated with START NOW as this is a manual-based program with no flexibility for the addition of other psychotherapy techniques or modification. START NOW consists of 32 unique sessions. Each group engaged in one session weekly, beginning with session one and moving consecutively through the lessons in the treatment manual. The psychotherapy utilized in the TAU groups is variable and draws from a variety of group-based psychotherapy techniques including but not limited to supportive therapy, twelve-step program, psychoeducation, and harm reduction.

Additionally, three physician-run groups were supported by postgraduate year three psychiatry residents with each resident assigned to a respective supervising attending physician. From this point forward, these physicians and NPs will be referred to collectively as psychotherapy group leaders or clinicians. All group leaders involved in this investigation were trained in START NOW and certified per the SN protocol prior to the initiation of the trial. Most group leaders ran both SN and TAU groups. A maximum number of 10 participants were allowed per group. Both SN and TAU involved a one hour once-weekly group-based psychotherapy administered for 32 weeks or 8 months. In the instance of federal holidays or inclement weather resulting in canceled group sessions, the START NOW curriculum and TAU was paused for that week and resumed the following week. In the instance that participants missed groups, they were allowed to continue attending group psychotherapy sessions after the initial 32-week period.

### Measures:

Participants’ age, gender, and self-reported race and ethnicity were collected from their electronic medical records.

The four primary endpoints include: (1) retention in treatment, which also translates to number of groups or weeks of psychotherapy attended, (2) days to last group attended, (3) intensity of attendance, and (4) drug screens. Attendance records were kept throughout the study, including date of consent, date of last group attended, and total number-of-groups-attended. Derived data fields were calculated from the attendance records: retention time (number of days from consent to last group attended) and attendance intensity (total number-of-groups-attended divided by number of weeks from consent to last group attended). Participants that did not attend any groups were assigned zero number-of-groups-attended and zero weeks-to-last-group; with regards to this data, there were no missing data to consider. Drug use during trial period was determined by drug screens, which were collected weekly or monthly based on clinician preference throughout the entire 8-month investigation for each participant via urine, blood, or salivary drug screens (Quest Diagnostics). Because tests were only collected based on clinician discretion, there is variability with the number of tests each individual received throughout the study.

All study data was collected and managed using REDCap electronic data capture tools hosted by Carilion Clinic (20). Due to the COVID-19 pandemic, our trial was terminated prematurely before achieving the targeted enrollment goal of 200 participants. The decision to terminate our study was directly because all group therapy sessions were halted indefinitely according to institutional policy to mitigate the COVID-19 transmission.

### Statistical Analysis:

Participant characteristics and clinical factors were described using means, standard deviations (SDs), medians, interquartile ranges (IQRs), frequencies, and percentages. Comparisons of continuous variables (age, number of groups attended, treatment duration, and intensity) by treatment group were performed using a two-sample t-test or non-parametric Wilcoxon two-sample tests, depending on normality. Comparisons of categorical variables (gender, race, and ethnicity) relied on chi-squared tests. Additionally, Cohen’s d (small: 0.20, medium: 0.50, large: 0.80) and Cramer’s V (small: 0.10–0.39, medium: 0.40–0.50, large: >0.50) effect sizes for continuous and categorical variables, respectively, were included for all comparisons (21). Side-by-side boxplots to visually compare the treatment groups regarding retention time outcomes (number of groups attended, treatment duration) were included.

Generalized linear models specifying a negative binomial distribution and log link were used to regress retention time outcomes on treatment group (primary effect of interest), while adjusting for age and gender. Similarly, a general linear model for intensity was generated by regressing the outcome on treatment group, gender, and age.

A post-hoc exploratory survival analysis was performed. Survival analysis is performed on data with at least two components: an event (yes vs. no) and time to that event. Here, the event is last group attended and time to event is the number of weeks between consent date and date of last group attended. Consequently, the event is confounded with time to event. Additionally, the survival curve will be equivalent to the empirical distribution of time to last group attended given that full information is available for each participant. As such, the analysis is exploratory, and the plots are provided as a complement to the generalized linear modeling. Time to last group was estimated using Kaplan-Meier (KM) methodology, with comparisons by treatment group accomplished using log-rank tests (22). Multivariable Cox proportional hazards modeling was used to explore the effects of treatment on time to last group attended, when adjusting for gender, and age group.

Drug screen analysis was performed using a generalized estimating equations (GEE) modeling framework specifying a binomial distribution and log link to examine changes in the odds for positive drug screens by treatment group over time, adjusting for age and gender. An independent covariance structure was used. Predictor variables included treatment group, week, a quadratic term for week (week x week), the interaction between treatment group and week (primary effect of interest), with age and gender serving as covariates. Participants who received at least one drug screen were included in this analysis. All statistical analyses were performed using SAS V9.4 (SAS Institute Inc., Cary, NC, USA). Statistical significance was taken at the p < 0.05 level and did not adjust for multiple comparisons.

## RESULTS

### Participants and Treatment Cohorts:

**Figure 1:**
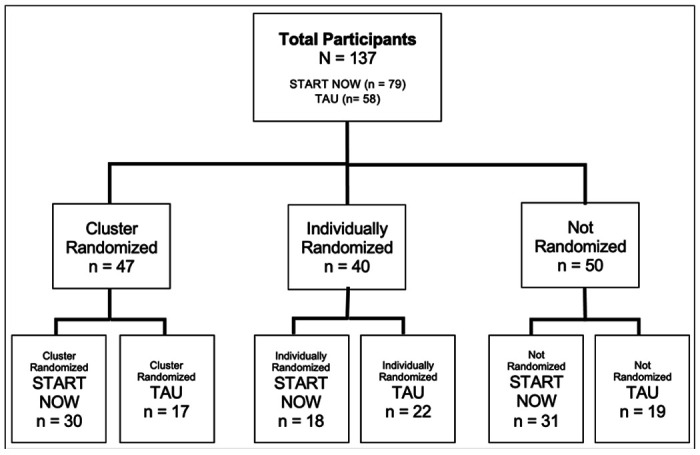
Participant Enrollment and Randomization Diagram

From November 2018 to February 2020, a total of 137 participants were quasi-randomized into START NOW (n = 79) or TAU (n = 58) for an approximately 8-month psychotherapy treatment duration (Fig. 1). Due to the COVID-19 pandemic, in-person psychotherapy was halted along with early termination of the trial, and therefore, there was no additional long-term follow-up performed beyond this timepoint. In total, there were 6 different weekly START NOW groups and 5 TAU groups with an average of 6–10 participants in each group respectively. Forty-seven participants were cluster-randomized with other participants resulting in 30 of these individuals in START NOW and 17 in TAU. Forty participants were individually randomized resulting in 18 of these individuals in START NOW and 22 in TAU. Fifty participants were not randomized with 31 participants enrolling into START NOW and 19 enrolling into TAU.

**Figure 2:**
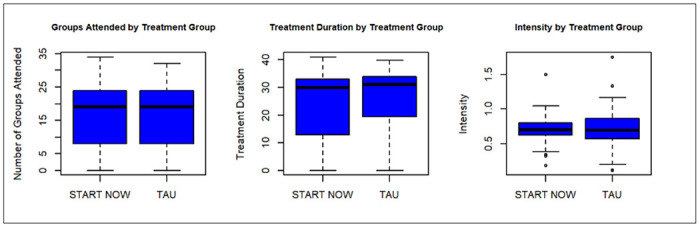
Boxplots for Retention Variables by Treatment Group

Multiple group leaders ran at least 1 START NOW group and 1 TAU group with a total of 8–10 facilitators (including psychiatry residents in the PGY3 year). Table 1 summarizes baseline participant characteristics by treatment group at time of consent. Participants in this sample were mostly female (59.1%), white (92.0%), and non-Hispanic (97.1%), with a mean age of 38.0 years (SD: 10.9). These results are consistent with the participant population in a pilot study (Truong et al., 2021). There was no statistically significant differences in race (p = 0.3992) and ethnicity (p = 0.4808) between the START NOW and TAU groups. Statistically significant differences in age (p = 0.0297, Cohen’s d[d] = 0.409), and gender (p = 0.0269, Cramer’s V[V] = 0.189) were observed between the treatment groups. Specifically, START NOW participants were significantly younger (mean age: 36.3 vs. 40.3 years) and more likely to be female (67.1% vs. 48.3%) compared to TAU participants at time of consent. Given these differences between the treatment groups at time of consent, all modeling was adjusted for age and gender. Boxplots to visually compare the retention time outcomes are included in Fig. 2.

### Treatment Effectiveness: Retention Time Outcomes

Table 2 summarizes all modeling results for all participants adjusting for age and gender. Model 1 shows that there were no statistically significant differences in the number of groups attended between START NOW and TAU participants when adjusting for age and gender (Incidence Rate Ratio [IRR] = 1.01, 95% Confidence Interval [CI] = 0.79, 1.28, p = 0.9541). The estimate for treatment in Model 2 shows treatment duration was 4% less in START NOW versus TAU participants (though not statistically significant), when adjusting for age and gender (IRR = 0.96, 95% CI = 0.77, 1.21, p = 0.7427). Model 3 demonstrates no differences in intensity between the treatment groups when adjusting for age and gender (Estimate=−0.01, Standard Error [SE] = 0.04, p = 0.8628).

**Figure 3:**
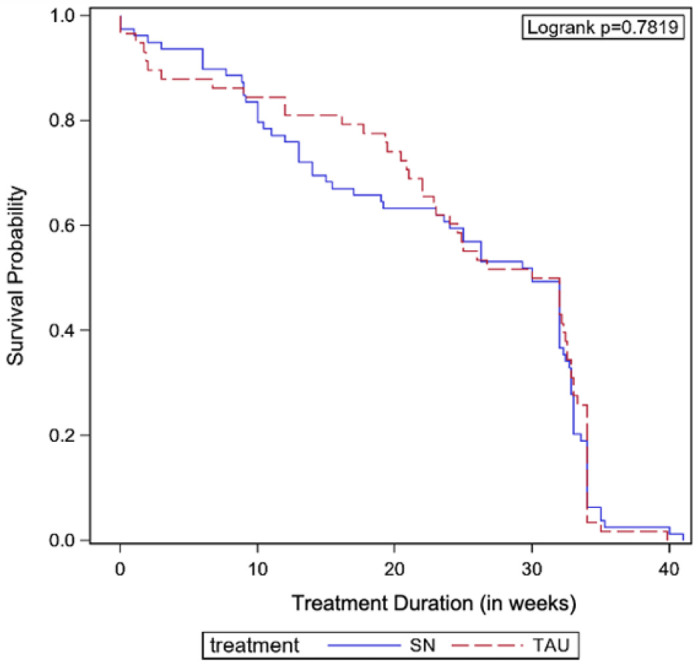
Kaplan-Meier Survival Curves by Treatment Group

The Kaplan-Meier curves including all participants for treatment duration (in weeks) by treatment group are shown in Fig. 3. No statistically significant difference by treatment group was observed (log-rank p = 0.7819). Similar results were shown when adjusting for age and gender using a multivariable Cox proportional hazards model (Table 2; Hazard Ratio [HR] = 1.01, 95% CI = 0.70–1.44, p = 0.9713).

### Treatment Effectiveness: Drug Screens

Out of 137 participants, only 135 participants provided at least one drug screen over the course of the trial. 2 participants enrolled 2 weeks prior to the COVID-19 shutdown and therefore did not provide any drug screens. Furthermore, as mentioned previously, drug screen data was inconsistently and sporadically collected because timing and frequency of drug screens was left to clinician discretion and failure of a participant to undergo drug screening does not preclude them from receiving treatment and therapy. Overall, participants enrolled in START NOW were not more likely than those in TAU to have any positive drug screen result (when excluding buprenorphine/naloxone) (Table 1; **81**% vs. 91.4%, p = 0.16). As seen in Table 3, further stratification of drug screen results over time based on week in therapy (from week 1 through week 40) also demonstrates no statistical significance with any positive drug screen between START NOW vs. TAU. Generalized estimating equations (GEE) modeling specifying a binomial distribution and log link was used to examine changes in the odds of positive drug screens by treatment group over time. Table 4 summarizes the model results for any positive drug screens. START NOW is associated with higher odds of a positive drug screen when compared to TAU (Odds Ratio [OR] = 1.21, 95% CI = 0.52–2.89, p = 0.6511), although not statistically significant. For every additional week in the program, the odds of a positive drug screen decreased by 1.4% (OR = 0.99, 95% CI = 0.92–1.06, p = 0.6914), but was not statistically significant. Additionally, there were no significant differences in changes in the odds for positive drug screens by treatment group over time when adjusting for all other variables in the model (Treatment x Week OR = 1.02, 95% CI = 0.98–1.07, p = 0.3475).

## DISCUSSION

Based on this quasi-randomized non-inferiority clinical trial, START NOW is noninferior to TAU–the standard of care–for treating OUD in a buprenorphine/naloxone medications for opioid use disorder office-based opioid treatment setting. START NOW psychotherapy has comparable rates of retention in treatment, days to last group attended, intensity of attendance, and positive drug screen testing. This suggests that adapted START NOW is an effective treatment modality for OUD.

As described previously, treating OUD requires evidence-based psychotherapy to be paired with MOUD. But because there is no gold standard treatment program, low-cost, effective programs that consider the whole patient (medical, psychosocial, socioeconomic factors) need to be developed and implemented (11, 12). Originally applied in the forensic population, START NOW represents a holistic, broad-based skills training program that has clinical effectiveness in the substance use patient population in which co-morbidities are common (23).

Data also suggests that skills-training may be particularly helpful for treating substance use disorders (24, 25). With START NOW, two skills are emphasized; focusing skills and the ABC (Activator, Behavior, Consequence) model for functional analysis of behavior which are key central tenets of this psychotherapy (13). Similarly, other investigations and reviews have found or argued that comprehensive approaches are most effective, and more studies are required (12, 26–28).

We believe that START NOW is a clinically effective psychotherapy program for the OUD patient population. Because START NOW is always free, it may be especially useful in low-resource settings, which have limited access to OUD treatment (29–31). Furthermore, a preliminary pilot study of START NOW for OUD conducted at our institution suggested that patients favorably view the skills-based training aspects START NOW and the diversity of the skills-training provided (14). This is important because patients need confidence in their treatment program which includes not only the clinician but also the psychotherapy provided.

This quasi-randomized clinical trial of START NOW for opioid use disorder patients undergoing buprenorphine/naloxone MOUD in an outpatient program represents a “real world” study design. Again, non-randomization occurred if a participant could only attend treatment at a specific time on a specific day–precluding flexibility for randomization. The rationale of this quasi-randomization method was to perform a study consistent with real-world addiction treatment and in situ limitations (32, 33). Our aim was to create an investigative model for studying a new psychotherapy in an active clinical setting. Often, these settings are filled with inherent challenges that may preclude the ability to perform a true individually randomized controlled trial. Furthermore, demonstrating non-inferiority within a pragmatic study demonstrates its application in clinical practice and provides greater generalizability to the broader clinical setting (34). As such, the aim of this study was to demonstrate noninferiority, showing that adapted START NOW provides clinical and research utility for the OUD patient population–a group that is inherently challenging to study (35).

As a result of the quasi-randomization method, this trial is limited as an effectiveness trial–rather than an efficacy trial–due to its design and early termination due to the COVID-19 pandemic. Interpretation of drug screen data is limited as a result of the sporadic nature by which these tests were conducted; drug screening occurred at regular intervals for some individuals (weekly), less frequently as determined by group leaders, or on individual’s own willingness to undergo consistent testing. As a result of the missingness and inconsistent data, the sporadic drug screen data is ultimately challenging to study and interpret.

While we aimed to enroll a total of 200 participants, post-hoc power calculations demonstrated that the achieved enrollment of 137 did not compromise power for the retention outcomes analyzed in this paper. However, limitations include early termination of the study and failure to perform long-term follow-up due to the start of the COVID-19 pandemic.

Our study population also was relatively homogenous (Caucasian, middle-age, female). While this may be viewed as a potential limitation, together with START NOW’s application in other patient populations such as the incarcerated population, adolescent girls, etc., we further demonstrate the general applicability of START NOW across different patient populations. We hypothesize that different populations may benefit more with an adapted form of START NOW psychotherapy that uses anecdotes and skills training examples that are specifically more relatable to their sex, ethnicity/race, and even culture. As previously mentioned, START NOW is customizable and adaptable across different populations, which is why it has been applied to correctional facilities, forensic psychiatric hospitals, and even a female adolescent population with oppositional defiant or conduct disorder (18).

Possible future studies include evaluating START NOW psychotherapy for treating OUD in patient populations with different patient demographics, socioeconomic backgrounds, and geopolitical factors. Future areas of research also includes evaluating START NOW’s effects on comorbid mood disorders like depression and anxiety within the OUD population. Additional areas of improvement for future studies includes consistent, mandatory drug screen collection and enhanced adaptations of START NOW specifically to each gender, age group, etc., thereby allowing the material to be even more applicable for each individual and for special populations (26, 36, 37).

Based on our experiences and the data available about trends in OUD, we believe that integrated, comprehensive interventions–centered around skills-training–are needed to effectively treat individuals with OUD. Based on the results of our trial, we propose that adapted START NOW, which is free for public use and therefore is accessible for healthcare professionals in low-resource settings, is an effective psychotherapy for treating OUD. START NOW psychotherapy should be considered an effective tool in settings, such as our own in Southwest Virginia that is disproportionately affected by the opioid crisis, in need of OUD psychotherapy for a patient population with extensive existing comorbidities and psychosocial challenges.

## CONCLUSION

Adapted START NOW is an effective psychotherapy for treating OUD when paired with buprenorphine/naloxone medications for opioid use disorder in the outpatient group therapy setting. Always free and publicly available, START NOW psychotherapy, along with its clinician manual and training materials, are easily accessible and distributable and may be especially useful for low-resource settings in need of evidence-based psychotherapy. Modification of START NOW to adapt teaching materials to be more culturally relevant may enhance its effectiveness.

## Figures and Tables

**Table 1:** Characteristics at Time of Consent by Treatment Group (N = 137)

	Overall Sample (N = 137)	START NOW (N = 79)	TAU (N = 58)	P-value^^^	Effect Size***
***DEMOGRAPHIC VARIABLES***					
AGE (YEARS)				**0.0297**	0.409 (Small)
n	137	79	58		
Mean ± SD	38.01 ± 10.904	36.32 ± 9.11	40.33 ± 10.84		
Median (Q1, Q3)	36.00 (31.00, 44.00)	34.00 (30.00, 42.00)	39.50 (31.00, 48.00)		
Min, Max	(18.00, 69.00)	(18.00, 69.00)	(22.00, 65.00)		
GENDER, n (%)				**0.0269**	0.189 (Small)
Female	81 (59.1%)	53 (67.09%)	28 (48.28%)		
Male	56 (40.9%)	26 (32.91%)	30 (51.72%)		
RACE, n (%)				0.3992	0.172 (Small)
Black	4 (2.9%)	3 (3.80%)	1 (1.72%)		
White	126 (92.0%)	73 (92.41%)	53 (91.38%)		
Native American or Alaskan Native	2 (1.5%)	0 (0.00%)	2 (3.45%)		
Other	1 (0.7%)	1 (1.27%)	0 (0.00%)		
Declined to Answer	4 (2.9%)	2 (2.53%)	2 (3.45%)		
ETHNICITY, n (%)				0.4808	0.103 (Small)
Hispanic	1 (0.7%)	0 (0.00%)	1 (1.72%)		
Non-Hispanic	133 (97.1%)	77 (97.47%)	56 (96.55%)		
Declined to Answer	3 (2.2%)	2 (2.53%)	1 (1.72%)		
*CLINICAL VARIABLES*					
NUMBER OF GROUPS ATTENDED				0.8041	0.038 (Small)
n	137	79	58		
Mean ± SD	16.53 ± 9.17	16.67 ± 9.22	16.33 ± 9.17		
Median (Q1, Q3)	19.00 (8.00, 24.00)	19.00 (8.00, 24.00)	19.00 (8.00, 24.00)		
Min, Max	(0.00, 34.00)	(0.00, 34.00)	(0.00, 32.00)		
TREATMENT DURATION*				0.6161	0.072 (Small)
Mean ± SD	24.13 ± 11.42	23.79 ± 11.56	24.61 ± 11.32		
Median (Q1, Q3)	30.00 (14.00, 33.00)	30.00 (13.00, 33.00)	31.00 (19.43, 34.00)		
Min, Max	(0.00, 41.00)	(0.00, 41.00)	(0.00, 39.86)		
INTENSITY**				0.9040	0.009 (Small)
n	133	77	56		
Mean ± SD	0.71 ± 0.24	0.71 ± 0.20	0.71 ± 0.29		
Median (Q1, Q3)	0.70 (0.59, 0.83)	0.70 (0.63, 0.80)	0.70 (0.57, 0.87)		
Min, Max	(0.12, 1.75)	(0.19, 1.50)	(0.12, 1.75)		
ANY POSITIVE DRUG SCREEN (EXCEPT BUP/NAL), n (%)	117 (86.7%)	64 (83.1%)	53 (91.4%)		
Yes	117 (85.4%)	64 (81.0%)	53 (91.4%)	0.1621	0.120 (Small)
No	18 (13.1%)	13 (16.5%)	5 (8.6%)		
Missing	2 (1.5%)	2 (2.5%)			

* Treatment duration is defined as the time from consent to last group attended in weeks.

** Intensity is number of groups attended divided by the number of weeks to last group.

*** Effect sizes are based on Cohen’s d (Small: 0.2, Medium: 0.5, Large: 0.8) for continuous variables and Cramer’s V (small: 0.10–0.39, medium: 0.40–0.50, large: >0.50) for categorical variables.

^ P-values are based on non-parametric Wilcoxon rank-sum tests for continuous variables and chi-squared tests for categorical variables.

**Table 2:** Model Results Adjusting for Age and Gender, All Participants

	Treatment (SN vs. TAU)	Age at Consent	Gender (Female vs. Male)
Model 1: Negative Binomial Model			
NUMBER OF GROUPS ATTENDED (N = 137)			
Estimate (IRR*)	1.0071	1.0010	1.1200
Estimate P-value	0.9541	0.8682	0.3487
IRR (95% CI)	(0.7915, 1.2814)	(0.9892, 1.0130)	(0.8836, 1.4197)
Model 2: Negative Binomial Model			
TREATMENT DURATION (N = 137)			
Estimate (IRR*)	0.9630	1.0019	1.1054
Estimate P-value	0.7427	0.7386	0.3797
IRR (95% CI)	(0.7692, 1.2058)	(0.9907, 1.0133)	(0.8840, 1.3822)
Model 3: General Linear Model			
INTENSITY (N = 133)			
Estimate (SE)	−0.0074 (0.0429)	−0.0017 (0.0021)	−0.0120 (0.0434)
Estimate P-value	0.8628	0.4178	0.7827
95% CI	(−0.0914, 0.0766)	(−0.0059, 0.0024)	(−0.9071, 0.0731)
Model 4: Cox Proportional Hazards Model			
TREATMENT DURATION (N = 137)			
Estimate (HR**)	1.0070	0.9930	0.8960
Estimate P-value	0.9713	0.4208	0.5393
HR (95% CI)	(0.7040, 1.4400)	(0.9750, 1.0110)	(0.6300, 1.2730)

* IRR = Incidence Rate Ratio. For example, the IRR for treatment in Model 1 is 1.0071. This means that START NOW participants will have a rate of group attendance 1.0071 times greater than TAU participants when age at consent and gender are held constant.

** HR = Hazard Ratio. For example, the HR for Treatment in Model 4 is 1.0070. This means that START NOW participants will have a risk of dropout 1.0070 times greater than TAU participants when age at consent and gender are held constant.

**Table 3:** Any Positive Drug Screen Results (except BUP/NAL) by Treatment Group over Time (N = 135)

Any positive drug screen (except BUP/NAL), n (%)	Overall Sample	START NOW	TAU	p-value	Effect size
Week 1	36 (94.74%,n = 38)	23 (100%,n = 23)	13 (86.7%,n = 15)	0.0720	0.292
Week 2	32 (94.1%,n = 34)	18 (94.7%,n = 19)	13 (86.7%,n = 15)	0.4101	0.141
Week 3	35 (100%,n = 35)	19 (100%,n = 19)	16 (100%,n = 16)	--	--
Week 4	36 (94.7%,n = 38)	19 (90.5%,n = 21)	17 (100%,n = 17)	0.1911	0.212
Week 5	29 (93.6%,n = 31)	15 (88.2%,n = 17)	14 (100%,n = 14)	0.1845	0.238
Week 6	39 (95.1%,n = 41)	20 (95.2%,n = 21)	19 (95%,n = 20)	0.9718	0.006
Week 7	27 (96.4%,n = 28)	16 (100%,n = 16)	11 (91.7%,n = 12)	0.2396	0.222
Week 8	31 (96.9%,n = 32)	15 (100%,n = 15)	16 (94.1%,n = 17)	0.3399	0.169
Week 9	33 (97.1%,n = 34)	16 (100%,n = 16)	17 (94.4%,n = 18)	0.3386	0.164
Week 10	33 (100%,n = 33)	17 (100%,n = 17)	16 (100%,n = 16)	--	--
Week 11	22 (95.7%,n = 23)	14 (93.3%,n = 15)	8 (100%,n = 8)	>*.9999*	0.156
Week 12	27 (93.1%,n = 29)	15 (93.8%,n = 16)	12 (92.3%,n = 13)	0.8788	0.028
Week 13	26 (96.3%,n = 27)	13 (100%,n = 13)	13 (92.9%,n = 14)	0.3261	0.189
Week 14	27 (93.1%,n = 29)	15 (100%,n = 15)	12 (85.7%,n = 14)	0.1292	0.282
Week 15	22 (95.7%,n = 23)	11 (91.7%,n = 12)	11 (100%,n = 11)	0.3276	0.204
Week 16	23 (92.0%,n = 25)	10 (90.9%,n = 11)	13 (92.9%,n = 14)	0.8586	0.036
Week 17	31 (93.9%,n = 33)	14 (87.5%,n = 16)	17 (100%,n = 17)	0.1326	0.262
Week 18	20 (100%,n = 20)	14 (100%,n = 14)	6 (100%,n = 6)	--	--
Week 19	17 (94.4%,n = 18)	11 (100%,n = 11)	6 (85.7%,n = 7)	*0.3889*	0.304
Week 20	16 (94.1%,n = 17)	9 (90.0%,n = 10)	7 (100%,n = 7)	>*.9999*	0.209
Week 21	19 (95.0%,n = 20)	11 (100%,n = 11)	8 (88.9%,n = 9)	*0.4500*	0.254
Week 22	27 (96.4%,n = 28)	16 (100%,n = 16)	11 (91.7%,n = 12)	0.2396	0.222
Week 23	20 (100%,n = 20)	13 (100%,n = 13)	7 (100%,n = 7)	--	--
Week 24	26 (92.9%,n = 28)	13 (100%,n = 13)	13 (86.7%,n = 15)	0.1719	0.258
Week 25	20 (100%,n = 20)	14 (100%,n = 14)	6 (100%,n = 6)	--	--
Week 26	19 (100%,n = 19)	10 (100%,n = 10)	9 (100%,n = 9)	--	--
Week 27	21 (95.5%,n = 22)	9 (90.0%,n = 10)	12 (100%,n = 12)	0.2622	0.239
Week 28	18 (100%,n = 18)	8 (100%,n = 8)	10 (100%,n = 10)	--	--
Week 29	17 (94.4%,n = 18)	10 (90.9%,n = 11)	7 (100%,n = 7)	>*.9999*	0.194
Week 30	17 (100%,n = 17)	11 (100%,n = 11)	6 (100%,n = 6)	--	--
Week 31	20 (100%,n = 20)	10 (100%,n = 10)	10 (100%,n = 10)	--	--
Week 32	22 (100%,n = 22)	11 (100%,n = 11)	11 (100%,n = 11)	--	--
Week 33	14 (93.3%,n = 15)	11 (100%,n = 11)	3 (75.0%,n = 4)	*0.2667*	0.443
Week 34	2 (66.7%,n = 3)	0 (0%,n = 1)	2 (100%,n = 2)	*0.3333*	1.000
Week 35	2 (100%,n = 2)	2 (100%,n = 2)	0 (0%,n = 0)	--	--
Week 36	2 (100%,n = 2)	2 (100%,n = 2)	0 (0%,n = 0)	--	--
Week 37	1 (100%,n = 1)	1 (100%,n = 1)	0 (0%,n = 0)	--	--
Week 38	1 (100%,n = 1)	1 (100%,n = 1)	0 (0%,n = 0)	--	--
Week 39	1 (100%,n = 1)	0 (0%,n = 0)	1 (100%,n = 1)	--	--
Week 40	2 (100%,n = 2)	1 (100%,n = 1)	1 (100%,n = 1)	--	--

* Effect sizes are based on Cramer’s V (small: 0.10–0.39, medium: 0.40–0.50, large: >0.50).

^ P-values are based on chi-squared and Fisher’s exact tests (italicized in table), as appropriate.

**Table 4:** Generalized Estimating Equations (GEE) Model Results for Any Positive Drug Screen (N = 135)

	Treatment (SN vs. TAU)	Week	Week X Week	Treatment X Week	Age at Consent	Gender (Female vs. Male)
ANY POSITIVE DRUG SCREEN						
Estimate (OR*)	1.2199	0.9855	0.9995	1.0220	0.9933	1.3072
Estimate P-value	0.6511	0.6914	0.6421	0.3475	0.6637	0.4217
OR (95% CI)	(0.5153, 2.8878)	(0.9172, 1.0590)	(0.9972, 1.0017)	(0.9766, 1.0695)	(0.9640, 1.0237)	(0.6801, 2.5123)

* OR = Odds Ratio. For example, the OR for treatment is 1.2199. This means that START NOW participants will have a rate of any positive drug screen 1.2199 times greater than TAU participants when adjusting for all other variables in the model.

## Data Availability

The datasets generated and analyzed during the current study are not publicly available to protect sensitive health data and the privacy of the study participants but are available from the corresponding author on reasonable request and are deidentified appropriately.

## Abbreviations

ASAM: American Society of Addiction Medicine
OBOT: office-based opioid treatment
OUD: opioid use disorder
MOUD: medications for opioid use disorder
SAMHSA: substance Abuse and Mental Health Services Administration
SN: START NOW
SUD: substance use disorder
TAU: treatment-as-usual
WHO: World Health Organization
